# Microstructural Characteristics of Interfacial Zone in Asphalt Mixture Considering the Influence of Aggregates Properties

**DOI:** 10.3390/ma13112558

**Published:** 2020-06-04

**Authors:** Jing Hu, Qibo Huang, Ning Lou, Sang Luo

**Affiliations:** Intelligent Transportation System Research Center, Southeast University, Nanjing 211189, China; huangqibo@seu.edu.cn (Q.H.); 220193223@seu.edu.cn (N.L.)

**Keywords:** asphalt mixture, microstructures, interfacial zone, mechanical behavior, viscoelastic characteristics

## Abstract

The interfacial zone between aggregate particles and asphalt mortar presents a significant effect on the strength of an asphalt mixture. In this paper, basalt, limestone, and diabase were selected, and the influence of these aggregates on the mechanical characteristics and microstructures of the interfacial zone was investigated. Nanoindentation was employed to measure the change law of mechanical behavior in the region of the interfacial zone, and corresponding viscoelastic parameters were deduced; microstructural morphology was observed by scanning electron microscopy, and the effect of the mineralogical components on the interfacial zone was analyzed as well. Results show that the mechanical behavior of the interfacial transition zone is complicated. The modulus and hardness of asphalt mortar decrease with the increases in the aggregate surface distance, and then keep stable after the distance is greater than 40 μm. Both the relaxation time and dissipated energy ratio of the interfacial zone affected by the different aggregate types show a similar change law. These states indicate that aggregate types have little influence on the stress dissipation of asphalt mortar. However, creep compliances that quantify the ability of the deformation resistance show a significant difference; microstructure morphologies of the interfacial zone are affected by aggregates obviously, and micro pores present a different distribution and state in the interfacial zone.

## 1. Introduction

Asphalt mixture is widely used as a material for pavement construction and maintenance, and crack is a common distress that deteriorates the strength and durability of a pavement structure. For asphalt mixture, interface failure between the aggregate and asphalt mortar often occurs during vehicle loading due to the weak adhesive strength and complicated stress states. It increases the probability of crack significantly. The interfacial transition zone (ITZ) is defined as a micro interfacial zone nearby the aggregate surface. It presents an obvious change trend of mechanical behavior and causes crack distress frequently. Therefore, investigating the mechanical characteristics and micromorphology of the ITZ is important to understand the fracture mechanism of the interfacial zone. Meanwhile, it is beneficial to effective damage evaluation and prediction. However, traditional technology cannot effectively measure and analyze the interfacial zone due to its microscale size. 

The aggregate plays an important role in the performance of an asphalt mixture. It affects the development of crack distress significantly [1,2]. Since micro-level test equipment was employed for concrete research in recent years, the morphology and mechanical behavior of the interfacial zone have been investigated to evaluate its damage characteristics. With the aim of analyzing the performance changes law of different materials, nanoindentation is widely used to measure the mechanical characteristics of asphalt mortar and mixture [3,4,5], and results of modulus and hardness provide accurate parameters for material evaluation, indicating nanoindentation is an effective method that detects a material’s mechanical characteristics, even for a material that distributes in an extremely small-scale region [6,7]. In addition, the relationship between the mechanical characteristics and microstructures can be established, and the strain rate-dependent compressive response of an asphalt mixture is characterized [8]. Besides the mechanical characteristics detected using nanoindentation, concrete’s microstructural morphology and components are important to investigate the material failure, especially the interfacial zone fracture. Although optical microscopy is an effective test equipment that observes the material components distribution of asphalt mixture [9], a microstructure observation needs more precise technology to identify the nano-scale substances. Nowadays, atomic force microscopy (AFM) and scanning electron microscopy (SEM) have been employed widely to explore the nano/micro-scale states of asphalt mortar and mixture. Utilizing AFM and SEM improves the research progress of interfacial zone characteristics because analyzing components and morphology are beneficial to understand the influence of microstructure on damage states [10,11,12,13,14,15]. Besides, to consider the influence of aggregate properties, mineralogical and chemical components of aggregates are also determined by X-ray diffraction (XRD) and X-ray photoelectron spectroscopy (XPS), respectively [16,17]. Thus, the relationship between the interfacial adhesion properties of asphalt mortar and aggregates with a different chemical and mineralogical nature was established [18,19].

The ITZ is a sensitive area that fractures easily due to vehicle loading. Its mechanical behavior affects the structural strength and durability of asphalt mixture obviously, therefore determining the mechanical parameters of the ITZ is beneficial to the crack distress evaluation of asphalt mixture. Presented researches have indicated that the range of the ITZ is from 30 to 60 μm near the aggregate surface [20,21]. Therefore, detecting the mechanical behavior of the ITZ is difficult and complex because of the microscale range. Actually, due to the notion that the nanoscale probe made of a known material can be used to measure unknown properties of a small volume testing sample, nanoindentation was employed to determine the mechanical characteristics change law of the interfacial zone across aggregate asphalt mortar through multiple indent points [22]. Viscoelastic parameters are calculated using the depths of the indent penetration, and a numerical model can be established to simulate the fracture process of the interfacial zone [23,24]. Researchers have developed a meso-scale numerical simulation based on a coupled plasticity–damage model to investigate the effect of asphalt mortar, aggregates, and the interface on the strength and damage response of concrete. This cohesive zone element is accepted to simulate the ITZ [25,26,27]. To determine the viscoelastic parameters based on the indent penetration, linear spring and dashpot elements are used to represent the creep behavior of a wide range of materials according to the hold period force–displacement data of nanoindentation test. Then, mechanical characteristics are described using viscoelastic mechanical models such as the Burgers and Maxwell models [28,29]. Besides, relation time and creep compliance are used to provide quantitative indicators to evaluate viscoelastic material properties [30,31].

It is well known that aggregate lithology has a significant influence on adhesion with asphalt mortar, such as limestone has a more powerful adhesive strength than basalt. In view of the aggregate particle, both chemistry and morphology were proved to affect the interfacial adhesive strength significantly [32,33,34]. This indicates that the interfacial zone failure is affected by aggregate lithology as well. However, although there is plenty of literature focus on the interfacial zone, researches related to influence of aggregates with different lithology on mechanical characteristics and morphology are few. Therefore, three aggregate types, basalt aggregates (BA), limestone aggregates (LA), and diabase aggregates (DA), were selected to determine the lithology discrepancy, quantifying the influence of the aggregate on the interfacial zone. Nanoindentation, XRD, and SEM were employed to detect the viscoelastic characteristics, mineralogical composition, and microstructure morphology of the interfacial zone, respectively. Then, the influence of the aggregates on the ITZ was analyzed.

## 2. Materials and Methods

### 2.1. Aggregate Investigation and Samples Preparation

Basalt, limestone, and diabase aggregates are common mineral materials used for pavement construction and maintenance. They show different adhesive performance with asphalt mortar that is important for structural strength. Table 1 shows the material properties of the aggregates used in this paper.

**Figure 1:**
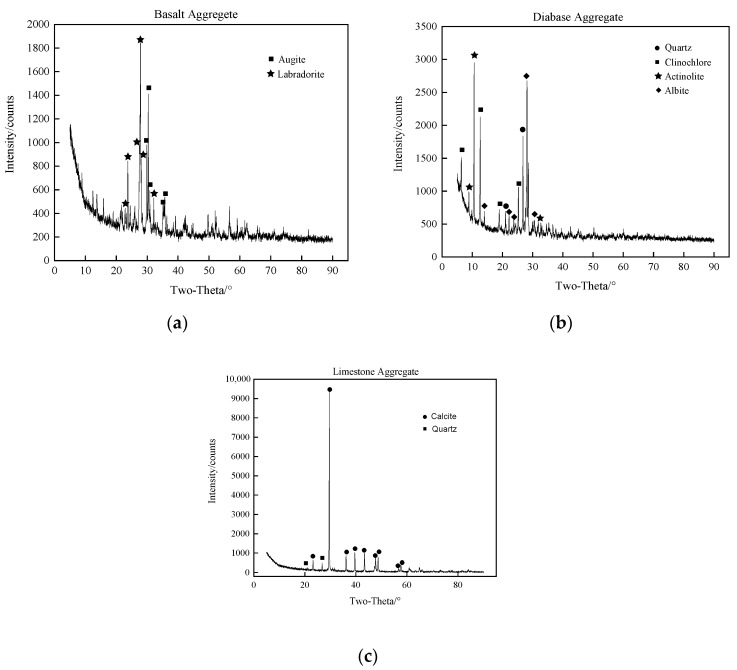
Intensity of mineralogical components of aggregates. (**a**) Basalt aggregates (BA), (**b**) diabase aggregates (DA) and (**c**) limestone aggregates (LA).

Aggregates can be divided into coarse and fine aggregates according to their sizes, and asphalt mortar is composed of fine aggregates, mineral power, and asphalt binder. Presented kinds of literature indicate that both an aggregate’s mineralogical components and chemical constitutions present a significant influence on the physical properties of an asphalt mixture, causing a different adhesive performance of the interface even if the aggregate has a similar volume and shape [35,36]. Since asphalt mortar consists of fine aggregates, mineralogical components also affect the microstructural characteristics and mechanical properties of the interfacial zone. To understand the mineralogical components and their corresponding proportions, X-ray Diffraction (XRD) was employed to detect the mineralogical components. The aggregate was ground and a powder whose size was less than 0.075 mm was collected. Test results of the XRD are illustrated in Figure 1 and detailed proportions are listed in Table 2.

Figure 1 illustrates that basalt and diabase contain more mineralogical components compared with limestone. Labradorite and augite are two main mineralogical components of basalt both in terms of weight and volume. Clinochlore and albite are two main mineralogical components of diabase, while limestone is almost composed of calcite. According to the hardness state of the mineralogical components, in theory, basalt shows the highest hardness, the next is diabase, and the hardness of limestone presents the lowest value. 

**Figure 2:**
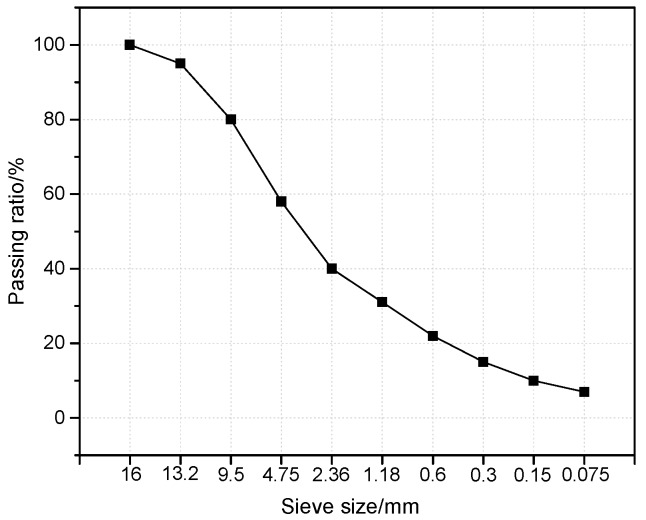
Gradation curve for the sample preparation.

As mentioned above, the interfacial zone is a contacting area between the coarse aggregate and asphalt mortar. Fine aggregate is the main component of asphalt mortar. Therefore, in order to assure the compatibility of the test results, asphalt mortar should be composed of fine aggregate according to the same size proportion. Therefore, samples containing different aggregates were prepared using the same gradation, as shown in Figure 2.

Bitumen modified by Styrene-Butadiene-Styrene triblock copolymer (SBS) was used as a binder to produce the asphalt mixture. Its properties are listed in Table 3. The Marshall method was employed to determine the optimal bitumen content of the asphalt mixture, and the content of 4.9% was accepted. 

A Superpave gyratory compactor with a vertical pressure of 600 kPa was employed to compact the asphalt mixture. The sizes of the cylindrical sample were 150 mm in diameter and 115 mm in height. 

### 2.2. Nanoindentation Theory

**Figure 3:**
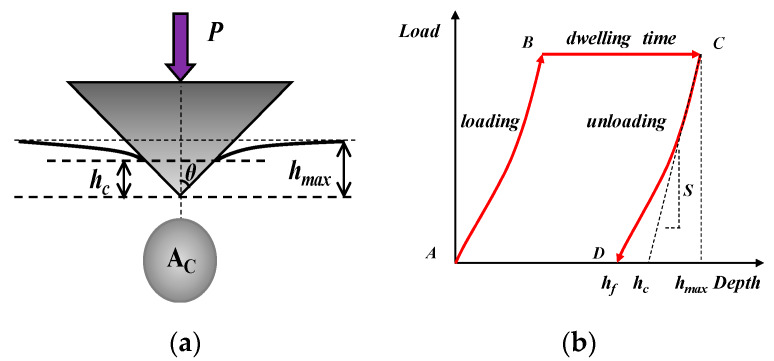
Nanoindentation test. (**a**) Geometry of the Berkovich tip, (**b**) typical nanoindentation load–depth curve.

The nanoindentation equipment manufactured by Micro Materials Ltd (Wrexham, UK) applies indention tip penetration using an electrostatic actuation and measures the probe depth by a capacitive sensor. The equipment can select either the load-controlled or depth-controlled mode [22]. Indent tip types are important to affect the probe depth, two indent tips are mainly utilized to penetrate the material in nanoindentation [37], namely a Berkovich tip and a spherical tip, and the presented literature indicates that the Berkovich tip is more suitable than the spherical tip for an asphalt binder. Therefore, the Berkovich tip was applied in this paper. The geometry of the nanoindentation test with a Berkovich tip and the typical indentation load (P)–depth (h) curve are shown in Figure 3.

Due to the viscoelasticity and creep behavior of asphalt materials, the indenting depth increases during the unloading process, leading to a negative slope in the unloading curve. According to Tarefder, introducing and extending the dwelling time can eliminate this effect on the unloading process by releasing the creep deformation of the asphalt mortar [38]. In this study, nanoscale indentation was applied on the surface of the aggregate and asphalt mortar, which consists of loading, extended dwelling time, and unloading stages. A load–depth curve was recorded to evaluate the mechanical properties of elastic or viscoelastic materials through the reduced modulus and hardness value derived from the Oliver–Pharr analysis method [39]. On the basis of the initial part of the unloading curve, the reduced modulus (*E_r_*) and the hardness (*H*) of the materials can be calculated by Equations (1) and (2):

(1) $$E_{r}=\frac{1}{\beta }\times \frac{S}{2}\times \frac{\sqrt{\pi }}{\sqrt{A_{C}}}$$

(2) $$H=\frac{P_{max}}{A_{C}}$$

where *S* is the contact stiffness determined from the initial slope in the unloading curve; *h_max_* is the maximum indentation depth; *β* is a geometrical correction factor for the Berkovich tip used in this study, *β* = 1.034; *P_max_* is the maximum indentation load; and *A_c_* is the projected contact area of the indent tip at maximum indentation load, and it is a function of the contact depth (*h_c_*) by polynomial fitting in the analysis software:

(3) $$A_{C}=-2000+2600h_{c}+24h_{c}{}^{2}$$

(4) $$h_{c}=h_{max}-0.75\frac{P_{max}}{S}$$

The absolute elastic modulus (*E_s_*) of test sample is solely responsible for the indentation depth recovery during the unloading process. The elastic modulus of the indenter (*E_i_*) is assumed known, so the *E_s_* can be determined using the given relations:

(5) $$\frac{1}{E_{r}}=\frac{1-v_{s}{}^{2}}{E_{s}}+\frac{1-v_{i}{}^{2}}{E_{i}}$$

(6) $$E_{s}=\left(1-v_{s}{}^{2}\right)\times {\left[\frac{1}{E_{r}}-\frac{1-v_{i}{}^{2}}{E_{i}}\right]}^{-1}$$

where, *E* and *υ* are the Young’s modulus and Poisson’s ratio, respectively, the subscript of *s* corresponds to the sample, and *i* corresponds to indent tip. For the indenter used in this study, the elastic modulus is *E_i_* = 1140 GPa and the Poisson’s ratio is *υ_i_* = 0.07. Based on the presented literature [22], a different Poisson’s ratio of the sample has limited influence on the calculation of the elastic modulus (*E_s_*), and the suggested Poisson’s ratio (*υ_s_*) of the sample in this test is set as 0.3.

### 2.3. Mechanical Characteristics of Interfacial Zone

**Figure 4:**
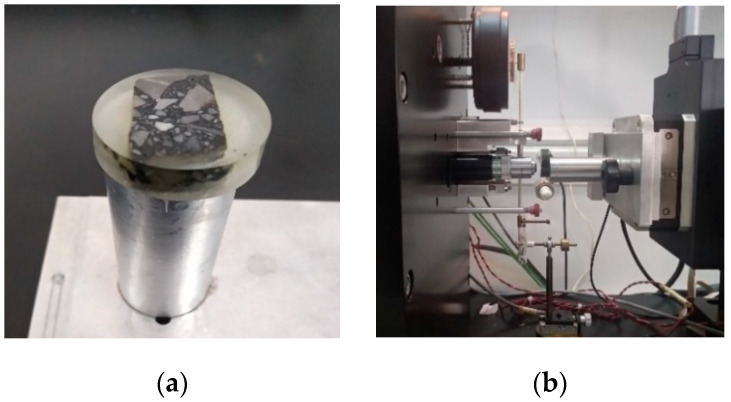
Sample used for the nanoindentation. (**a**) Test sample after the smoothing process, (**b**) the nanoindentation test.

Small cuboid samples whose sizes were 10.0 mm × 10.0 mm × 5.0mm were cut from test samples for the nanoindentation. Due to the nanoscale requirement of the measure, the roughness of the sample surface presents a significant influence on the indentation depth and mechanical properties [22]. Therefore, a smooth sample surface is necessary to ensure the test precision. To smooth the surface applied by the indent tip, the sample was placed in a cylindrical container and embedded by epoxy resin, and then demolded after epoxy resin solidification to facilitate the smoothing process, as shown in Figure 4a. The surface was smoothed using a grinding machine to achieve a 0.05 μm level of the smooth surface, and epoxy resin that covers the sample surface was removed to avoid it affecting the test results. The sample was measured by nanoindentation aimed at the distinct interfacial zone between the aggregate surface and asphalt mortar, as shown in Figure 4b. 

**Figure 5:**
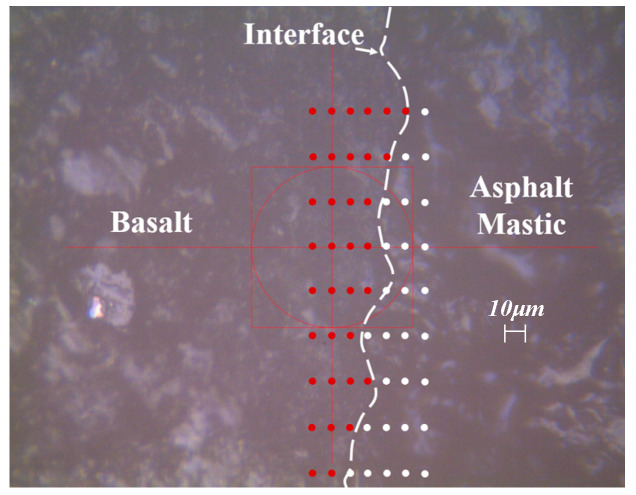
Distribution of indentation positions.

Grid indent points were carried out on the smooth surface using a NanoTest instrument (Micro Materials) equipped with a Berkovich tip, and the test temperature was 25 °C. To assure the interfacial zone was covered by grid indent points, the sample was observed using an optical microscope to select the interesting area and assign indent positions throughout the distinct phases of the aggregate and asphalt mortar. These indent points should cover the phases of the aggregates and asphalt mortar, as shown in Figure 5. For each sample, a total 7 × 9 indentations were set and tested to calculate the modulus and hardness value of the different phases. The distance of each indentation was 10 μm to avoid the disturbance of neighboring indenting deformations. In this paper, the load-controlled mode was selected, the maximum load and loading rate and unloading rate were 0.3 mN, 0.01 mN/s, and 0.01 mN/s, according to previous literature [39]. Since the influence of viscoelastic properties can be decreased by applying an extended dwelling time between the connective indentations [38], 200 s was applied to fully release the creep deformation of the asphalt mortar after reaching the maximum load. The initial load was 0.05 mN to facilitate the contact between the indent tip and sample surface. 

### 2.4. Investigation on Morphology of Interfacial Zone

**Figure 6:**
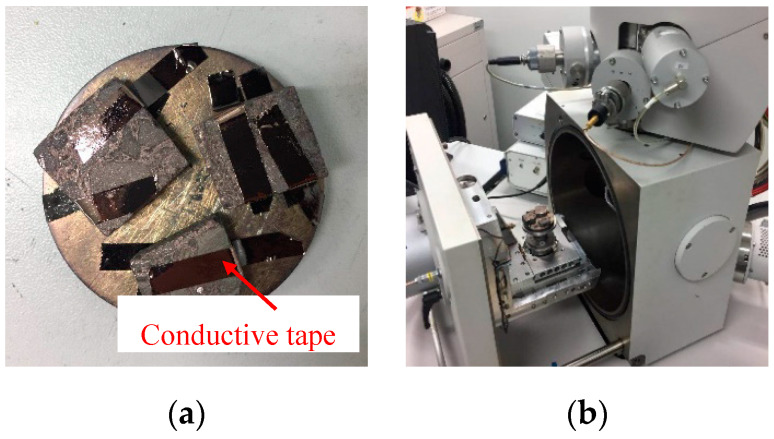
Morphology investigation using SEM. (**a**) Test samples for SEM, and (**b**) SEM equipment.

Morphology features are important factors that affect the mechanical behavior of the interfacial zone, a complex adhesive interface, and the micro voids structure increases the probability of an interface fracture. Scanning electron microscopy (SEM) manufactured by FEI Ltd (Hillsboro, OR, USA) was used to provide images of the interfacial zone based on the secondary electron (SE) and backscattered electron (BSE) imaging techniques, so that the spatial morphology and micro voids distribution could be observed, respectively. Test samples used for the SEM observation were cubes whose sizes were 15.0 mm × 15.0 mm × 5.0mm, and the outer surface of the sample was sprayed by a gold film and adhered conductive tape to increase conductivity, as shown in Figure 6. Using SEM equipment, we searched for a suitable image of the interfacial zone, and different magnifications of 500, 1500, and 2000 times were conducted to observe in more detail. 

## 3. Results and Discussion

### 3.1. Mechanical Properties of Interfacial Zone

**Figure 7:**
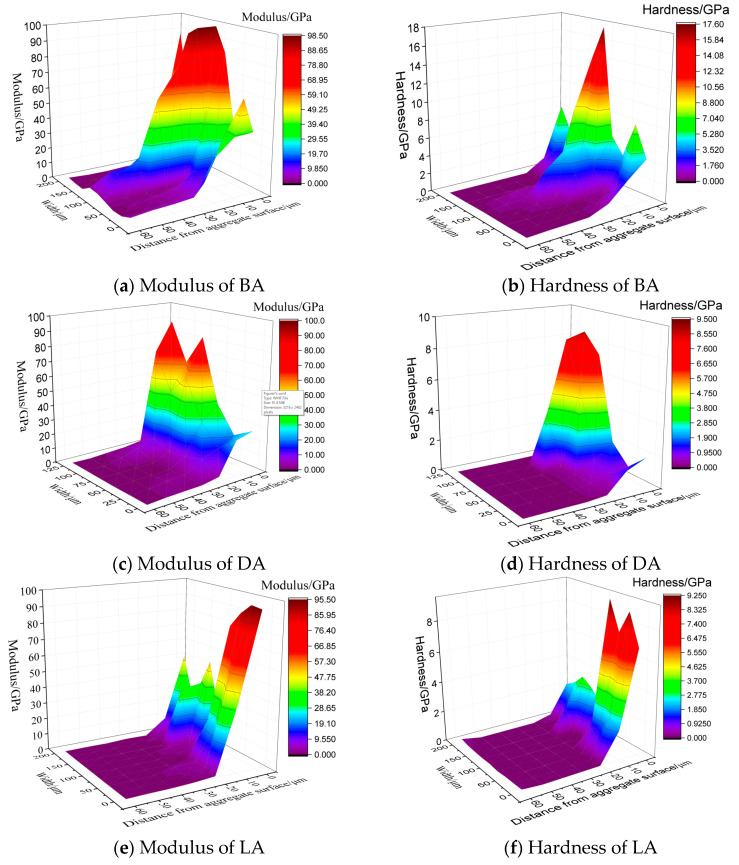
Modulus and hardness distribution of the interfacial zone. (**a**) and (**b**) BA, (**c**) and (**d**) DA, and (**e**) and (**f**) LA.

Crack distress appears at the interface zone easily because of the stress concentration of micro pores and the complex stress field affected by the aggregate and asphalt mortar; therefore, the mechanical properties of the interface zone are important for evaluating the strength and durability of an asphalt mixture. However, it is hard to determine an accurate value of the elastic modulus and hardness that quantifies the mechanical behavior of the interfacial zone. Some of the presented literature have indicated that the mechanical properties of the interfacial zone change gradually nearby the aggregate surface [7,21]. Actually, the ITZ can be regarded as a transition region of the mechanical properties from the aggregate to the asphalt mortar. Nanoindentation detects the modulus and hardness of the indent points covering the interfacial zone, and spatial distributions of these two parameters are shown in Figure 7. 

It should be noted that the indentation points at a distance of 0 μm do not cover the aggregate at all because of the zigzag surface. However, at least one indentation point acts on the aggregate. Figure 7 shows the modulus and hardness distribution according to the distance from the aggregate surface. It can be concluded that influences of the aggregate on the modulus and hardness of the asphalt mortar are significant. The adhesion effect with the aggregate surface increases the modulus and hardness of the asphalt mortar. In general, both the modulus and hardness decrease rapidly with the increases in distance and then remain a stable value. Therefore, the complicated mechanical properties of the asphalt mortar nearby the aggregate surface lead to the complex stress field of this region. 

**Figure 8:**
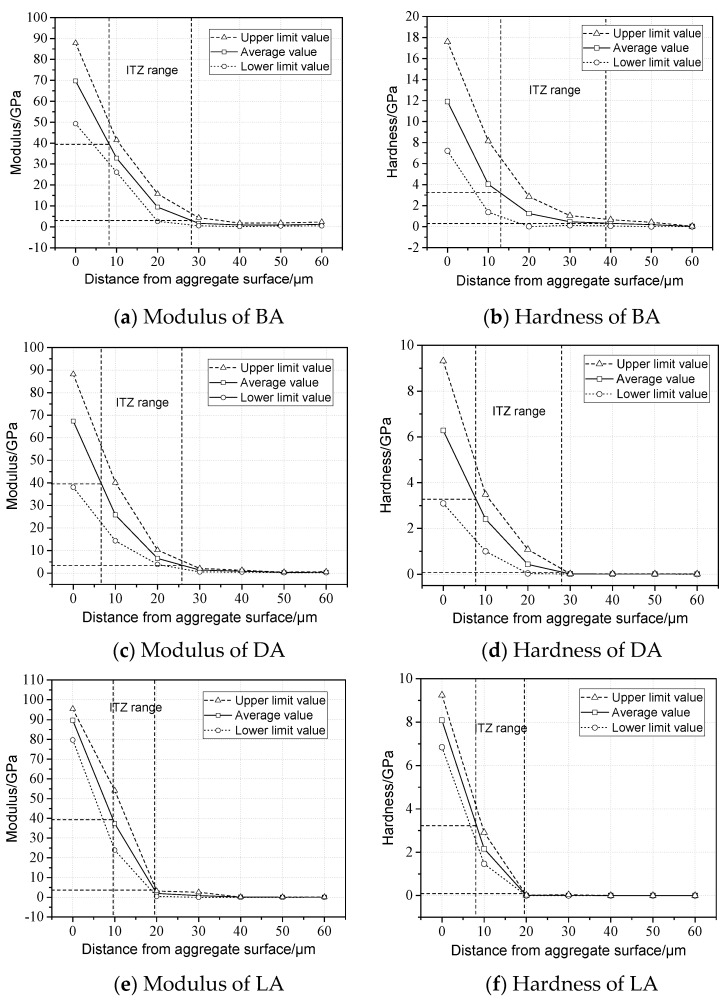
Modulus and hardness versus distance. (**a**) and (**b**) BA, (**c**) and (**d**) DA, and (**e**) and (**f**) LA.

To quantify the change law of the mechanical properties of the interfacial zone and evaluate the ITZ range, the modulus and hardness versus the distance from the aggregate surface were plotted, as shown in Figure 8. 

Figure 8 illustrates the changing trend of the modulus and hardness versus the distance from the aggregate surface, where the modulus of all the aggregate types decreases rapidly with the increase in distance from the aggregate surface. Figure 8a shows that the modulus decreases mainly at the range of 20 μm and then decreases slowly until the distance of 40 μm, indicating that the influence of the aggregate on the modulus of the asphalt mortar can be neglected if the distance is greater than 40 μm. The influence range of DA is supposed to be 40 μm. Similarly, influence ranges of DA and LA are approximately equal to 30 and 20 μm, respectively. For hardness, Figure 8b,d,f show that the hardness presents similar trends with that of the modulus, and the affect ranges of BA, DA, and LA are 40, 30, and 20 μm. According to a conclusion that the modulus and hardness of the ITZ are 3.1 to 39.9 GPa and 0.005 to 3.2 GPa, respectively [21], the descending order of the ITZ range of BA, DA, and LA is shown in Figure 8. Therefore, it is concluded that the influence of the aggregates on the mechanical properties is significant, where on the one hand, adhesion with the coarse aggregate surface improves the modulus and hardness of the interfacial zone, and on the other hand, fine aggregates affect the mechanical behavior of the asphalt mortar obviously. Besides, the ITZ range of LA is less than that of BA and DA, indicating that simple mineralogical components and less hardness may reduce the ITZ range.

The modulus and hardness quantify the distribution of the interfacial zone’s mechanical properties, therefore parameters describing the viscoelastic behavior of the interface should be determined as well. The maximum depths of the indentations during the dwell time were used to evaluate the viscoelastic characteristics of the aggregate and asphalt mortar.

**Figure 9:**
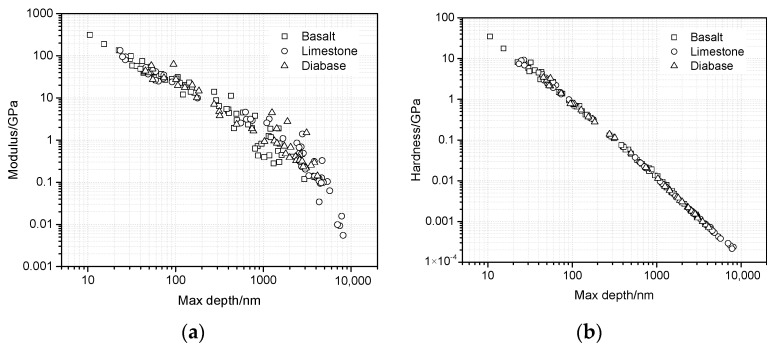
Modulus and hardness versus maximum depth. (**a**) Modulus, (**b**) hardness.

Figure 9 illustrates that the modulus and hardness decrease with the increases in maximum depth at the dwell time of 200 s. A great maximum depth means a high percentage of viscoelastic deformation. Figure 9a shows the relationship between the modulus and maximum depth under logarithmic coordination. It can be observed that the modulus data that belong to different aggregates show the low correlation, especially when the maximum depth is greater than 100 nm. However, Figure 9b shows that the hardness data that belong to different aggregates present an almost linear correlation under a logarithmic relationship. Therefore, the hardness presents better relativity with the maximum depth compared with the modulus, and this reveals that asphalt mortars of the interfacial zone appear similar in final deformation if their hardness is similar.

**Figure 10:**
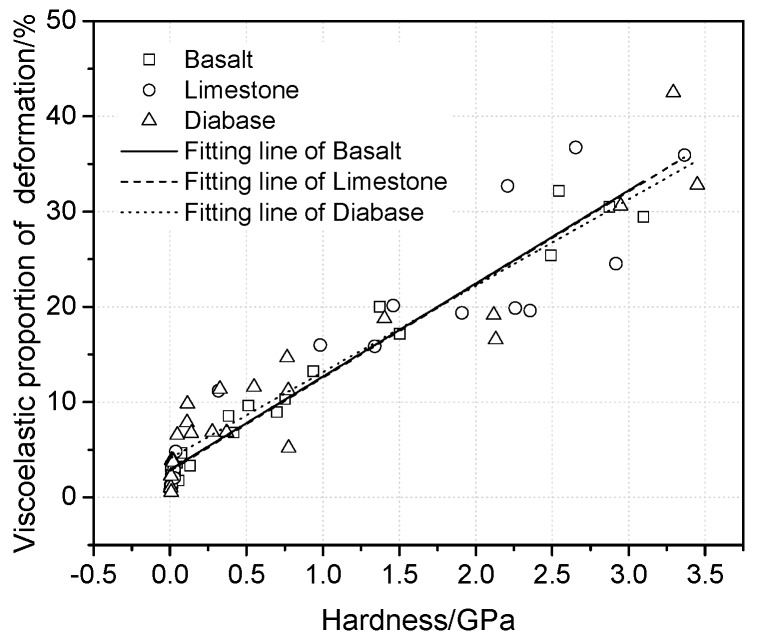
The relationship between viscoelastic proportion of deformation and hardness.

Based on discussions above, the maximum depth of the asphalt mortar includes viscoelastic and plastic deformation. The viscoelastic one is important to affect the mechanical behavior. Viscoelastic proportions of maximum depths were determined, and the relationship between the viscoelastic proportion and hardness was analyzed, as shown in Figure 10.

Within the hardness range of the ITZ [7,21], the viscoelastic proportion of the depth increases with the increases in hardness. This phenomenon indicates that asphalt mortar with high hardness presents more viscoelastic deformation under the same deformation. As the hardness is gradually decreasing with the distance from the aggregates surface which is increasing, the viscoelastic proportion of the deformation decreases as well. In other words, asphalt mortar close to the aggregate surface presents significant viscoelastic characteristics and deformation recovering performance.

### 3.2. Viscoelasticity States of Interfacial Zone

**Figure 11:**
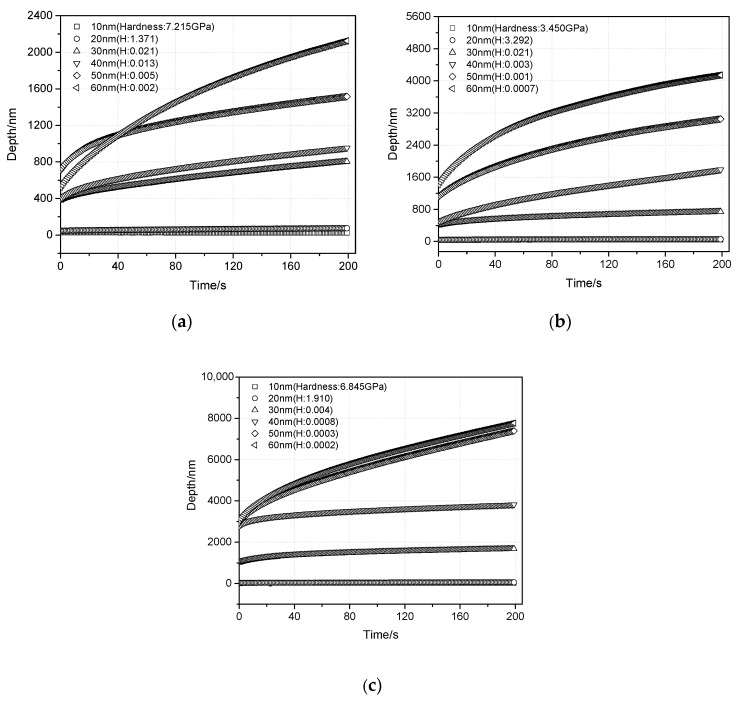
Creep curves of indentation points during the dwell time. (**a**) BA, (**b**) DA, and (**c**) LA.

Understanding the viscoelastic behavior of the ITZ is beneficial to investigate the fracture mechanism of the interfacial zone between the aggregate and asphalt mortar, and can provide mechanical parameters to determine the stress field using a numerical simulation. In the nanoindentation test, the load is kept constant during the dwell time of 200 s after it reaches the peak value of 0.03 mN. The creep curve can be used to deduce the viscoelastic characteristics of the interfacial zone. Figure 11 shows a typical change trend of the creep depth versus dwell time, where six indentation points whose distances from the aggregate surface are 10 to 60 nm are compared. 

Figure 11 shows a great difference in the creep depths of the different asphalt mortar. The depth increases with the increases in the distance from the aggregate surface due to the modulus and hardness decrease. Based on the discussion from Figure 10, a low hardness leads to a less viscoelastic proportion of deformation, namely a great plastic deformation that cannot recover. Besides, for the different aggregate types, the creep depth of the LA interfacial zone shows a larger value compared with that of BA and DA. This can be attributed to the low modulus and hardness.

Asphalt mortar can be simplified as a viscoelastic material. Thus, the Burgers model was used to describe the viscoelastic characteristics of the interfacial zone. The Burgers model is composed of the Maxwell and Kelvin models. They are two basic models reflecting the stress relaxation and rheological or creep, respectively [40,41]. According to the research results [28], the penetration depth of the indentation increases with the time given by the well-known Hertz equation with the addition of a time-dependent exponential, as shown in Equation (7).

(7) h2(t)=π2pocotα[1E1+1E2(1−e−tE2η2)+1η1t]

where *h(t)* are penetration depths of the asphalt mortar, in nm; *P_o_* is a constant load, in mN; *t* are times of the test, s; *E*_1_ is the instantaneous elastic modulus of the Maxwell model, reflecting the deformation capacity of the material, in MPa; *η*_1_ are viscous coefficients of the Maxwell model, indicating the anti-flow deformation capacity of the materials, in MPa·s; and *E*_2_ and *η*_2_ are the viscoelastic parameters that present the creep behavior, where the units are MPa and MPa·s.

These four Burgers indicators, including *E*_1_, *E*_2_, *η*_1_, and *η*_2_, were fitted using creep curves during the dwell time of the nanoindentation, and the results at the dwell time of 200s were used. The relaxation time is a quantitative indicator representing the stress change with time. It is important to evaluate the stress relaxing rate and stress dissipation property [40]. To determine the relaxation time (*λ*), the following equation is obtained.

(8) $$\lambda =\frac{\eta_{1}}{E_{1}}$$

In this paper, the average relaxation time of the indentation points was used to show the distribution in the interfacial zone. As mentioned above, relaxation time indicates the performance of stress dissipation; a smaller relaxation time means an excellent performance of the stress dissipation. 

**Figure 12:**
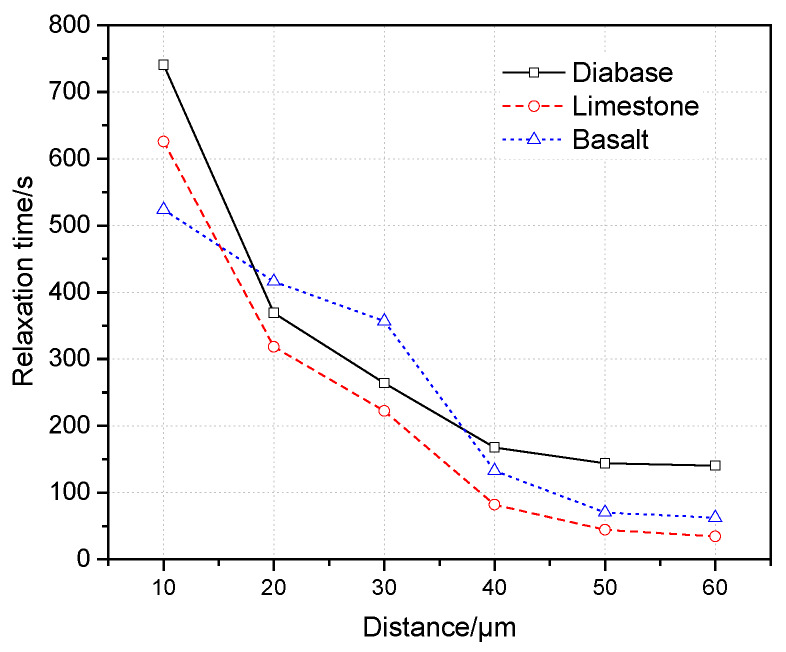
Relaxation time distribution in the interfacial zone.

Figure 12 illustrates that the relaxation time decreases with the increases in the distance from the aggregate surface, meaning the stress dissipation was improved away from the aggregate surface. In brief, although the three curves of the relaxation time are similar and intersecting, limestone’s interfacial zone shows a better performance of stress dissipation, indicating stress dissipation is affected by the aggregate type to some degree. It should be noted that the change rates of the relaxation time are great within the distance range from 10 to 40 μm, and then the relaxation time tends to be stable when the distance is greater than 40 μm. Therefore, the stress dissipation of the asphalt mortar increases rapidly with the increases in distance. It is beneficial to avoid a stress concentration; the influence of the aggregate surface on the stress dissipation is negligible after the distance is greater than 40 μm.

Considering the nature of the Burgers model components, where springs are associated with storage and dashpots with the dissipation of the deformation energy, the Burgers model can be used to indicate the asphalt mortar’s capacity of energy dissipation. In a mechanical point of the viscoelastic model, the ideal spring element and dashpot element in the Maxwell or Kelvin models simulate energy storage and consumption, respectively. The parameters of *E*, *η,* and *t* are used to indicate the capacity of the energy storage and dissipation [40]. Stored energy and dissipated energy are calculated by Equations (9) and (10).

(9) Ws(t)=σ02[1E1+12E2(1−2e−tE2η2+e−2tE2η2)]

(10) Wd(t)=σ02[1η1t+12E2(1−e−2tE2η2)]

where *W_s_(t)* is the stored energy per volume, in MPa, *t* is the loading time of the test, in s, *σ*_0_ is the maximum stress, in MPa, and *W_d_(t)* is the dissipated energy per volume, in MPa.

The dissipated energy ratio is the ratio of *W_d_(t)* to *W_s_(t)*. A large dissipated energy ratio means a good internal flow of the material and an excellent performance of the stress dissipation. Due to the notion that stored energy and dissipated energy are functions of time (t), the end of the dwell time of 200s was selected to determine the energy values. Besides, creep compliance is another performance criterion of a viscoelastic material, and therefore a great creep compliance means a weak deformation resistance. The formula of creep compliance can be drawn as Equation (11).

(11) J(t)=1E1+tη1+1E2(1−etE2η2)

where *J(t)* is the creep compliance, in 1/MPa.

**Figure 13:**
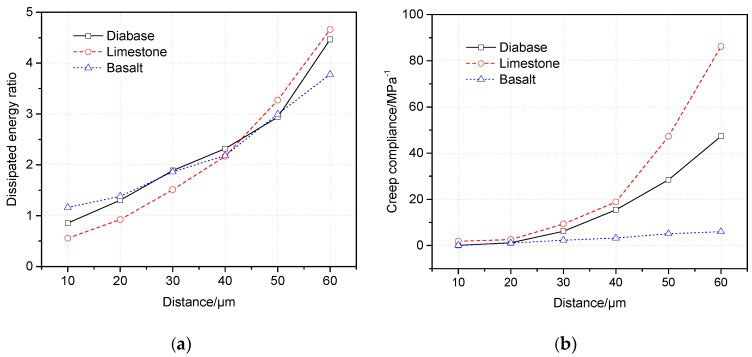
Dissipated energy ratio and creep compliance. (**a**) Dissipated energy ratio. (**b**) Creep compliance.

The dissipated energy ratio is similar to the relaxation time for indicating the stress dissipation, and all three curves in Figure 13a show that the dissipated energy ratio increases with the increases in distance. Therefore, performances of the internal flow and stress dissipation of the asphalt mortar are enhanced. The deduction is entirely consistent with the results of the relaxation time. For the creep compliance shown in Figure 13b, it increases with the increases in distance as well, indicating the deformation resistance decreased gradually. It is noteworthy that the creep compliances of the three interfacial zones show an obvious difference, where the interfacial zone of basalt presents a stable and great deformation resistance compared with limestone and diabase. 

**Figure 14:**
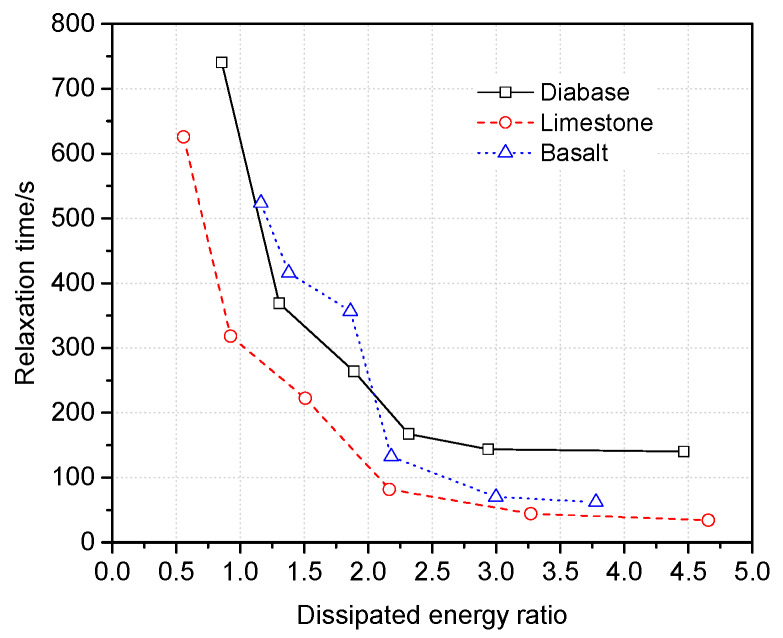
Relationship between the dissipated energy ratio and relaxation time.

According to the results of the presented literature that the relaxation time and dissipated energy ratio of an asphalt binder show a linear relationship, a large dissipated energy ratio correlates with less relaxation time [40]. Since relaxation times and dissipated energy ratios represent the relaxation property, their relationship in the asphalt mortar of the interfacial zone should be investigated. Figure 14 shows that relaxation time decreases with the increases in the dissipated energy ratio. The correlation is nonlinear because of the action of fine aggregates; the change rate decreases gradually, and then the change rate tends to stabilize. It should be noted that turning points of the change rate almost locate at 2.0 to 2.5 of the dissipated energy ratio, and the corresponding distances are 40 μm according to data of Figure 13a. Therefore, it can be assumed that the range of 40 μm from the aggregate surface is a sensitive area because of complicated mechanical characteristics. 

### 3.3. Microstructural States of Interfacial Zone

**Figure 15:**
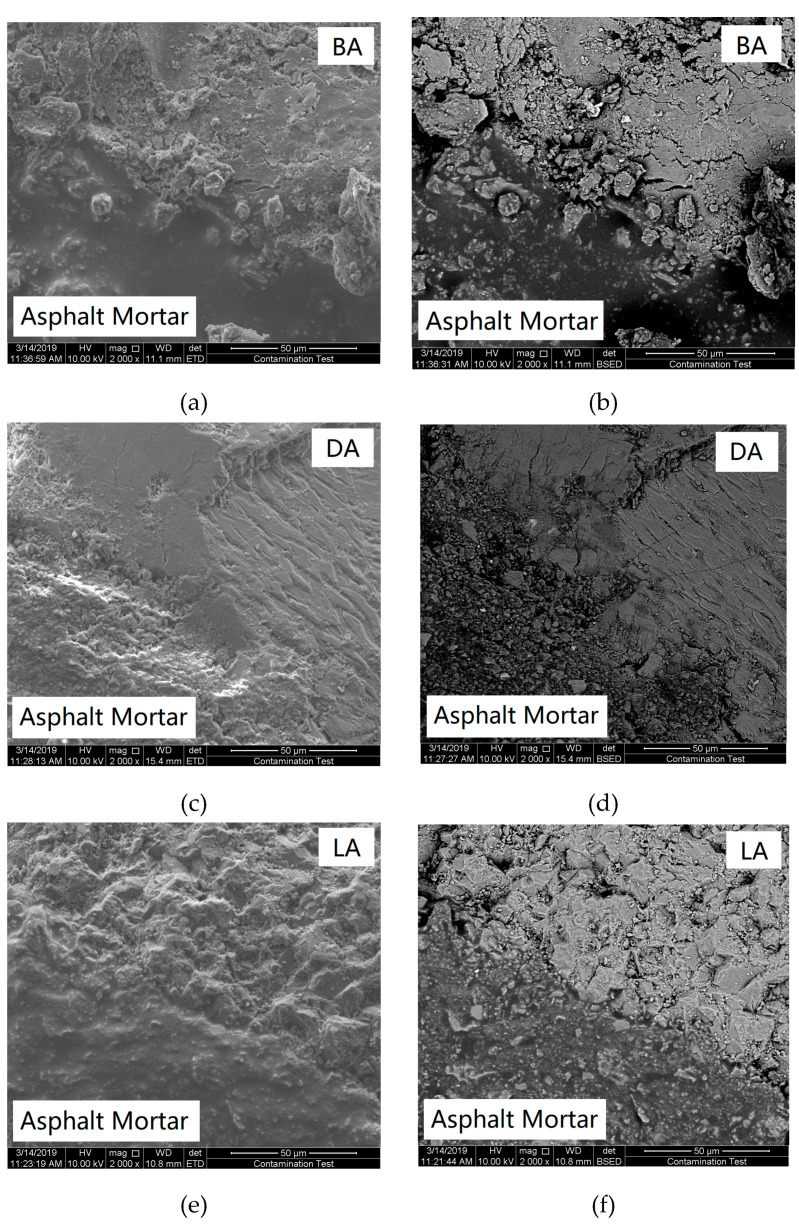
Microstructural states of the interfacial zone. (**a**) BA-SE, (**b**) BA-BSE, (**c**) DA-SE, (**d**) DA-BSE, (**e**) LA-SE, and (**f**) LA-BSE.

The microstructures of the interfacial zone present a significant influence on the crack distress, where a complicated morphological state aggravates the stress concentration, especially the tiny pores in the interfacial zone. In this paper, high-resolution images were obtained by an SEM device to observe the morphological state and pores distribution, and some typical images of the SE images and BSE images are shown in Figure 15, where the magnification was 2000 times.

**Figure 16:**
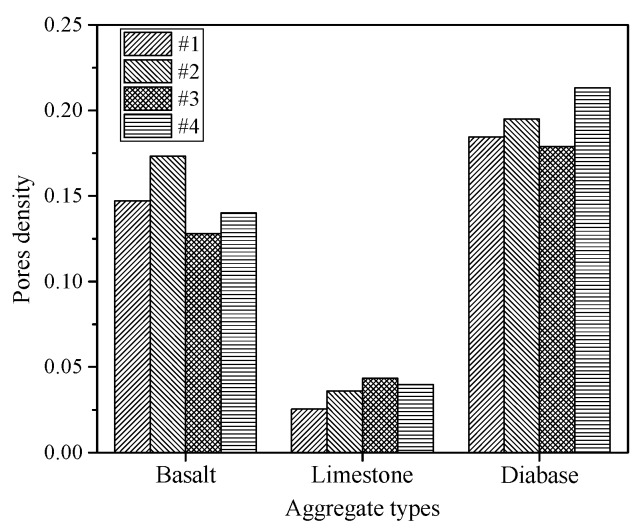
Pores density of the different interfacial zones.

Figure 15 shows the microstructural state of the interfacial zone between the asphalt mortar and aggregate surface. The SE and BSE images can be used to illustrate the apparent morphology and surface pores distribution, respectively. Pores mainly appear at the contact interface between the asphalt mortar and aggregate surface, indicating that the interface fractures easily due to the notion that pores deteriorate the adhesive strength of the interface. Besides, some small pores exist in the asphalt mortar nearby the aggregate, especially for diabase shown in Figure 15d, and these pores have an adverse effect on the mechanical performance of the interfacial zone. To quantify the pores state using a BSE image, an outline of the contact interface was delineated and its translated line was set to define a region used to calculate the pores state. The distance between these two lines was 40 μm based on the sensitive range. The BSE image was converted into a gray image, and image segmentation using the gray threshold was utilized to identify pores in this region; pixels that had a gray value less than 20 were classified as pores and the area was calculated. The pores density was defined as the ratio of the pores area to the region area, quantifying the pores proportion per unit area. Four samples of each aggregate type were analyzed, as shown in Figure 16. 

Figure 16 shows an obvious discrepancy in the pores density of the different interfacial zones, where the aggregates present an influence on the pores significantly. The pores density of the diabase and limestone interfacial zones presents the maximum and minimum values, respectively. Therefore, the pores decrease if limestone is used as the aggregates of the asphalt mixture, and decreasing pores reduce the probability of crack distress because the pores can cause initial cracks of the interface. Furthermore, pores probably affect the viscoelastic characteristics of the interfacial zone, and fewer pores may improve the stress dissipation and deformation resistance. 

## 4. Summary and Conclusions

The interfacial zone between an aggregate and asphalt mortar is a weak region in an asphalt mixture, and crack distress appears at this region due to vehicle load and deteriorates the strength of the asphalt mixture. In order to evaluate the influence of aggregates on the interfacial zone, the mechanical behavior was measured by nanoindentation, and the mineralogical components and microstructures were detected by XRD and SEM. The aggregate affects the mechanical behavior of the interfacial zone significantly, and the modulus and hardness of the asphalt mortar decrease with the increases in the distance from the aggregate surface. The influence of aggregate type on the ITZ range is obvious, limestone produces a smaller ITZ range compared with basalt and diabase, and 40 μm can be defined as the influence scope of the aggregate on the mechanical behavior of the interfacial zone.

For the different aggregate types, the hardness approximately shows a linear correlation with the maximum depth of the nanoindentation penetration and viscoelastic proportion of the deformation. This observation means that hardness is a better indicator that quantifies the deformation of the interfacial zone compared with the modulus. The relaxation time decreases with the increase in the distance from the aggregate surface, but the dissipated energy ratio and creep compliance show the opposite tendency. The results indicate that the stress dissipation and internal flow are improved with the distance from the aggregate surface, while the deformation resistance is reduced. In general, the stress dissipation and internal flow of the different aggregates are similar, indicating a similar ability of preventing a stress concentration. However, the deformation resistance of the interfacial zone nearby the basalt aggregate is greater than that of diabase and limestone. This is mainly because the mineralogical components of basalt have greater hardness.

Pores distribute in the interfacial zone, and mainly appear at the contact interface between the aggregate surface and asphalt mortar. These pores deteriorate the adhesive strength of the interfacial zone and increase the probability of crack distress. The aggregate types have a significant influence on the pores density, and using limestone as aggregate is beneficial to reduce pores in the interfacial zone.

It should be noted that bitumen is a temperature-sensitive material, and its mechanical behavior is affected by temperature significantly. However, the test temperature in this paper was 25℃ because of the restrictions of the test devices, although the influence of temperature on the ITZ will be investigated in the future research.

## Figures and Tables

**Table 1:** Material properties of different aggregates.

Aggregate Type	Apparent Relative Density (g/cm^3^)	Water Absorption/%	Crushing Value/%	Acicular Content/%
Basalt	2.946	0.51	9.6	0.7
Diabase	2.932	0.42	11.4	1.2
Limestone	2.881	0.86	10.4	5.8

**Table 2:** Mineralogical components of different lithological aggregates.

Aggregate Types	Mineralogical Components	Hardness/ Mohs	Desity/ g/cm^3^	Molecular Formula	Weight/%	Volume/%
Basalt	Labradorite	6.0~6.5	2.65~2.75	NaAlSi_3_O_8_ CaAl_2_Si_3_O_8_	85.7	88.2
	Augite	5.5~6.0	3.20~3.60	Ca(Mg,Fe,Al)[(Si,Al)_2_O_6_]	12.2	10.0
	Other	–	–	–	2.1	1.8
Diabase	Clinochlore	2.0~3.0	2.61~3.30	(Mg,Fe)_4.75_Al_1.25_(Al_1.25_Si_2.75_O_10_)(OH)_8_	63.9	63.7
	Albite	6.0~6.5	2.61~2.64	Na_2_O·Al_2_O_3_·6SiO_2_	18.9	20.6
	Other	–	–	–	14.4	12.8
Limestone	Calcite	2.7~3.0	2.60~2.80	CaCO_3_	95.6	95.5
	Other	–	–	–	4.4	4.5

**Table 3:** Properties of SBS-modified bitumen.

Specification	Unit	SBS-Modified Asphalt
Penetration (25 °C, 100 g, 5 s)	mm	5.4
Ductility (5 °C, 5 cm/min)	cm	27
Softening point	°C	74
Viscosity (135 °C)	Pa∙s	1.92
